# Glycogen synthase kinase-3β modulation of glucocorticoid responsiveness in COPD

**DOI:** 10.1152/ajplung.00077.2015

**Published:** 2015-08-28

**Authors:** Anta Ngkelo, Roland F. Hoffmann, Andrew L. Durham, John A. Marwick, Simone M. Brandenburg, Harold G. de Bruin, Marnix R. Jonker, Christos Rossios, Eleni Tsitsiou, Gaetano Caramori, Marco Contoli, Paolo Casolari, Francesco Monaco, Filippo Andò, Giuseppe Speciale, Iain Kilty, Kian F. Chung, Alberto Papi, Mark A. Lindsay, Nick H. T. ten Hacken, Maarten van den Berge, Wim Timens, Peter J. Barnes, Antoon J. van Oosterhout, Ian M. Adcock, Paul A. Kirkham, Irene H. Heijink

**Affiliations:** ^1^Airways Disease Section, National Heart and Lung Institute, Imperial College London, London, United Kingdom;; ^2^University of Groningen, University Medical Center Groningen, Department of Pathology and Medical Biology, Groningen, The Netherlands;; ^3^Medical Research Council Centre for Inflammation Research, Queen's Medical Research Institute, University of Edinburgh Medical School, Edinburgh, United Kingdom;; ^4^Respiratory Research Group, Faculty of Medical and Human Sciences, University of Manchester, and National Institute for Health Research Translational Research Facility in Respiratory Medicine, University Hospital of South Manchester, Manchester, United Kingdom;; ^5^Dipartimento di Scienze Mediche, Sezione di Medicina Interna e Cardiorespiratoria, Centro per lo Studio delle Malattie Infiammatorie Croniche delle Vie Aeree e Patologie Fumo Correlate dell'Apparato Respiratorio (formerly termed Centro di Ricerca su Asma e BPCO), Università di Ferrara, Ferrara, Italy;; ^6^Thoracic Surgery Unit, Cardiovascular and Thoracic Department, University of Messina, Messina, Italy;; ^7^Pneumology Unit, Cardiovascular and Thoracic Department, University of Messina, Italy;; ^8^Department of Human Pathology “Gaetano Barresi,” University of Messina, Messina, Italy;; ^9^Pfizer, Inflammation and Remodeling Research Unit, Cambridge, Massachusetts;; ^10^Department of Pharmacy and Pharmacology, University of Bath, Claverton Down, Bath, United Kingdom;; ^11^University of Groningen, University Medical Center Groningen, Department of Pulmonology, Groningen, The Netherlands; and; ^12^University of Groningen, University Medical Center Groningen, Groningen, Groningen Research Institute for Asthma Research Institute, Groningen, The Netherlands

**Keywords:** COPD, oxidative stress, inflammatory responses, monocytes, epithelial cells

## Abstract

In chronic obstructive pulmonary disease (COPD), oxidative stress regulates the inflammatory response of bronchial epithelium and monocytes/macrophages through kinase modulation and has been linked to glucocorticoid unresponsiveness. Glycogen synthase-3β (GSK3β) inactivation plays a key role in mediating signaling processes upon reactive oxygen species (ROS) exposure. We hypothesized that GSK3β is involved in oxidative stress-induced glucocorticoid insensitivity in COPD. We studied levels of phospho-GSK3β-Ser9, a marker of GSK3β inactivation, in lung sections and cultured monocytes and bronchial epithelial cells of COPD patients, control smokers, and nonsmokers. We observed increased levels of phospho-GSK3β-Ser9 in monocytes, alveolar macrophages, and bronchial epithelial cells from COPD patients and control smokers compared with nonsmokers. Pharmacological inactivation of GSK3β did not affect CXCL8 or granulocyte-macrophage colony-stimulating factor (GM-CSF) expression but resulted in glucocorticoid insensitivity in vitro in both inflammatory and structural cells. Further mechanistic studies in monocyte and bronchial epithelial cell lines showed that GSK3β inactivation is a common effector of oxidative stress-induced activation of the MEK/ERK-1/2 and phosphatidylinositol 3-kinase/Akt signaling pathways leading to glucocorticoid unresponsiveness. In primary monocytes, the mechanism involved modulation of histone deacetylase 2 (HDAC2) activity in response to GSK3β inactivation. In conclusion, we demonstrate for the first time that ROS-induced glucocorticoid unresponsiveness in COPD is mediated through GSK3β, acting as a ROS-sensitive hub.

chronic obstructive pulmonary disease (COPD) is characterized by chroniclung inflammation, airway remodeling, and pulmonary emphysema, which leads to airflow limitation and accelerated lung function decline. Current therapies fail to prevent either disease progression or mortality. Glucocorticoids are widely used because of their broad anti-inflammatory effects, but they provide relatively little therapeutic benefit in COPD (3). The reduced responsiveness to the anti-inflammatory effects of glucocorticoids is a major barrier to effective management of COPD patients. Therefore, there is an urgent need to understand the underlying molecular mechanisms.

The increased oxidant burden in the lungs of COPD patients, derived from cigarette smoke (CS) and the respiratory burst from inflammatory cells, plays a significant role in the reduced glucocorticoid responsiveness (4, 10, 25, 33, 41). Oxidative stress has a profound impact on inflammation by inducing proinflammatory mediators that attract and activate neutrophils, including CXCL8 and granulocyte-macrophage colony-stimulating factor (GM-CSF). This induction is driven by activation of redox-sensitive kinase pathways [including MAPK and phosphatidylinositol 3-kinase (PI3K)/Akt signaling] and proinflammatory transcription factors such as the nuclear factor-κB (NF-κB) (8, 26, 27, 31, 32, 37). In addition, oxidative stress can induce PI3K-dependent posttranslational histone deacetylase 2 (HDAC2) modifications, including phosphorylation, resulting in proteasomal HDAC2 degradation (1, 22, 27, 33). HDAC2 can deacetylate the glucocorticoid receptor (GRα) as well as histones at NF-κB response elements within promoter regions of inflammatory genes (21–23). Reduced HDAC2 expression has been observed in the lungs and alveolar macrophages of COPD patients and has been implicated in glucocorticoid insensitivity in COPD (22, 23). In addition to alveolar macrophages, we recently observed that bronchial epithelial cells from COPD patients are less responsive to glucocorticoids than those from healthy controls (16). Here, proinflammatory cytokine production was effectively suppressed by glucocorticoids in cells from healthy controls, while this response was compromised in COPD-derived cells.

The constitutively active serine/threonine kinase glycogen synthase-3β (GSK3β) is regulated by oxidative stress and has been linked to several inflammatory diseases (6, 20, 24). GSK3β activity is negatively regulated by phosphorylation on serine 9, which can be mediated by ERK1/2 MAPK and Akt (15). As these kinase pathways are commonly involved in oxidant-mediated responses, GSK3β may represent an important downstream effector of oxidant-mediated signaling during COPD inflammation. We hypothesized that GSK3β is involved in oxidative stress-induced glucocorticoid responsiveness in COPD. We demonstrate that levels of inactive GSK3β are enhanced in monocytes, macrophages, and bronchial epithelial cells from COPD patients compared with smokers and nonsmokers and that oxidative stress-induced GSK3β inhibition regulates glucocorticoid responsiveness in both monocytes/macrophages and bronchial epithelial cells.

## METHODS

### 

#### Human studies.

Peripheral lung sections, peripheral venous blood, primary bronchial epithelial cells (PBECs), and tissue sections were isolated from age-matched nonsmokers, smokers with normal lung function, and COPD patients. For GSK3β phosphorylation and total staining analysis, peripheral lung sections were collected from 21 patients with COPD, 19 smokers, and 14 nonsmokers subjects (Table 1) and from 12 COPD patients, 12 smokers, and 10 nonsmokers (Table 2), respectively. Phospho-GSK3β was also detected in tissue macrophages of severe COPD patients (Table 1). Peripheral venous blood was collected from 10 patients with COPD, 6 smokers with normal lung function, and 7 nonsmokers (Table 3). PBECs were obtained from 14 current and ex-smoking COPD patients with Global Initiative for Chronic Obstructive Lung Disease (GOLD) guideline classification stage II-IV, 16 age-matched control smokers, and 14 nonsmokers (Table 4) (17). With the exception of the COPD stage IV patients, subjects did not use inhaled corticosteroids, long-acting β-agonists, and long-acting anticholinergics for at least 4 wk preceding the study. The study protocol for this part was consistent with the Research Code of the University Medical Center Groningen (https://www.umcg.nl/SiteCollectionDocuments/English/Researchcode/UMCG-Researchcode,%20basic%20principles%202013.pdf) and national ethical and professional guidelines (*Code of Conduct; Dutch Federation of Biomedical Scientific Societies*; https://www.federa.org/codes-conduct).

**Table 1.** Characteristics of subjects for the immunohistochemical study of phospho-GS3K*β*

	Nonsmokers	Smokers	COPD	Severe COPD
Age	67.7 ± 8.1	70.0 ± 6.7	69.1 ± 6.6	70.3 ± 2.8
Sex (M/F)	0/14	18/1	18/3	7/0
Current/former smokers	N/A	9/10	7/14	3/4
Pack years	N/A	49.4 ± 32.3	40.5 ± 20.1	50.6 ± 11.6
FEV_1_, liter	2.1 ± 0.4	2.5 ± 0.7	2.03 ± 0.5	1.13 ± 0.073
FEV_1_, %pred	101.5 ± 22.5	91.8 ± 14.6	75.3 ± 16.6	41.3 ± 3.0
FEV_1_/FVC ratio, %	76.4 ± 3.5	75.5 ± 4.6	56.1 ± 9.1	51.7 ± 4.9
GOLD stage	N/A	N/A	8 Grade 1, 11 grade 2, 2 grade 3	All grade 3

Data are presented as means ± SD. Peripheral lung tissue sections were collected from patients recruited from the Section of Respiratory Diseases of the University Hospital of Ferrara. GS3Kβ, glycogen synthase-3β; FEV_1_, forced expiratory volume in 1 s; FVC: forced vital capacity. GOLD, Global Initiative for Chronic Obstructive Lung Disease guideline classification of patients with chronic pulmonary disease (COPD); %pred, %predicted; M, male; F, female. The FEV_1_/FVC ratio is after bronchodilator for subjects with COPD but not for smokers or nonsmokers.

**Table 2.** Characteristics of subjects for the immunohistochemical study of total GS3K*β*

	Nonsmokers	Control smokers	COPD
Age	69.1 ± 2.5	65.4 ± 1.9	69.5 ± 2.1
Sex (M/F)	2/8	12/0	12/0
Current/former smokers	N/A	6/6	7/5
Pack years	N/A	49.1 ± 12.1	37.9 ± 3.3
FEV_1_, %pred	111.2 ± 6.2	90.5 ± 4.8	70.4 ± 3.8
FEV_1_/FVC ratio, %	78.1 ± 1.4	76.8 ± 1.2	59.4 ± 2.1

Data are presented as means ± SD. Peripheral lung tissue sections were collected from patients recruited from the Section of Respiratory Diseases of the University Hospital of Ferrara.

**Table 3.** Characteristics of subjects: peripheral blood monocytes

	Nonsmokers	Controls smokers	COPD
Age	57 ± 4.4	55.5 ± 3.2	65.6 ± 4.4
Sex M/F	3/4	4/2	8/2
Current/former smokers	N/A	5/1	2/8
Pack years	N/A	31 ± 12.3	33.1 ± 14.2
FEV_1_, %pred	105.6 ± 7.5	92.2 ± 14.5	68.65 ± 16
FEV_1_/FVC ratio	72.54 ± 3.9	73.02 ± 8.8	58.79 ± 15.7

Data are presented as means ± SD. Peripheral venous blood was collected from patients at the Royal Brompton hospital of London.

**Table 4.** Characteristics of the subjects: primary bronchial epithelial cells

	Nonsmokers (*n* = 11)	Control Smokers (*n* = 12)	COPD (*n* = 8)
Age	56 (43–76)	54 (43–70)	56.5 (50–65)
Sex M/F	5/6	9/3	4/4
Pack years	0 (0–0)	39.5 (19–50)	33.5 (11–54)

Medians (range) or number. All experimental controls are included. Primary bronchial epithelial cells (PBECs) included in the study were obtained from Lonza or the NORM and TIP Study within the University Medical Center Groningen. COPD patients were included on a basis of FEV_1_<50% of predicted, FEV_1_/FVC <70%, and ≥10 pack years for GOLD stage IV. All control subjects had FEV_1_/FVC >70% and FEV_1_>90% of predicted. PBECs obtained from Lonza are not indicated with FEV_1_/FVC and FEV_1_ predicted or pack years.

All participants gave informed consent to a protocol approved by the ethics committee of the Royal Brompton and Harefield National Health Service Trust/National Heart and Lung Institute and the University Medical Center Groningen. The Section of Respiratory Disease at the University Hospital of Ferrara and the Pneumology Unit at the University Hospital of Messina have ethics committee approval for the collection and analysis of specimens from lung resection surgery.

#### Cell culture.

Peripheral blood mononuclear cells (PBMCs) were isolated from whole venous blood by Histopaque (Sigma, Dorset, UK), as previously described (27). Monocytes were isolated from PBMCs by MACS with the Monocyte Isolation Kit II (Miltenyi Biotec, Bergisch-Gladbach, Germany). PBECs were cultured in bronchial epithelium growth medium (BEGM; Lonza, Breda, The Netherlands) in flasks coated with collagen and fibronectin as described previously (19). Human bronchial epithelial (16HBE) cells were kindly provided by Dr. G. Gruenert and grown in EMEM/10% FCS (UCSF) (18).

#### Cell culture and treatments.

Isolated monocytes and human monocyte-macrophage cells (MonoMac6) were cultured in RPMI 1640 GlutaMAX phenol red free media (Invitrogen, Paisley, UK) with 1% FCS, 2 mM l-glutamine, 1% nonessential amino acids, and 1% sodium pyruvate. Primary monocytes were pretreated with U0126 (MEK/ERK-1/2 inhibitor), MK-2206 (Akt inhibitor), or IC87114 (PI3K-δ inhibitor) all at 1 μM for 30 min and were stimulated with hydrogen peroxide (H_2_O_2_; 100 μM) for 30 min. Primary monocytes were pretreated with the GSK3β inhibitor CT99021 (100 nM and 1 μM) for 15–120 min as indicated. To study the function of glucocorticoids, primary monocytes or transfected MonoMac6 cells were pretreated with dexamethasone (10 nM, 100 nM, and 1 μM) for 30 min before being stimulated with LPS (10 ng/ml) for 16 h. Cells or cell-free supernatants were harvested for RNA isolation, cell lysate preparation, or measurement of cytokines.

PBECs were cultured for at least 3 wk and used at passage 3. PBECs and 16HBE cells were passaged by trypsin, plated in 24-well plates, and grown to ∼90% confluence. Subsequently, PBECs were hormone/growth factor-deprived using basal medium (BEBM; Lonza) supplemented with transferrin and insulin (PBECs) and 16HBE cells were serum-deprived overnight.

PBECs and 16HBE cells were pretreated with or without 7.5% cigarette smoke extract (CSE) for 6 h. *N*-acetyl-cysteine (NAC; 5 mM) was added 90 min before CSE exposure. CT99201 (10 μM) was added 30 min before CSE treatment for 6 h or budesonide (1, 10, and 100 nM) treatment for 2 h and cells were subsequently stimulated with TNF-α (10 ng/ml) for 24 h. Cells or cell-free supernatants were harvested for RNA isolation, cell lysate preparation, or measurement of cytokines.

#### Transfections.

GSK3β on-target siRNA (Dharmacon) was used according to the manufacturer's instructions. The HA-GSK3β-S9A-pcDNA3 and HA-GSK3β-K85A-pcDNA3 expression vectors were kindly provided by Dr. J. Woodget (Toronto, Canada). The following plasmids were obtained from Addgene: plasmid 14754-GSK3β S9A mutant pcDNA3 (36); Addgene plasmid 14755-GSK3β K85A mutant pcDNA3.1; or the negative control pcDNA3 construct lacking the GSK3β insert (36).

#### Cigarette smoke extract.

Two 3R4F research cigarettes (Tobacco Research & Development Center, Lexington, KY) bubbled at 70 rpm through 25 ml EMEM, using a high flow peristaltic pump (Watson Barlow, Rotterdam, The Netherlands) represents 100% CSE. The extract was prepared freshly for each experiment.

#### Quantitative RT-PCR.

RNA was isolated and heme-oxygenase-1 (HO-1) mRNA expression was analyzed by real-time PCR using Taqman (Applied Biosystems, Foster City, CA) as described previously (16). Validated probe and housekeeping genes, β-2-microglobulin (B2M), and peptidylprolyl isomerase A (PP1A) and TaqMan Master Mix were purchased from Applied Biosystems.

#### Lung tissue processing and immunohistochemistry.

Lung tissue processing and immunohistochemistry were performed as previously described (44). The anti-phospho-GSK3β-Ser9 (sc-11757-R) and anti-total GSK3β (sc-9166) antibodies were obtained from Santa Cruz Biotechnology (Heidelberg, Germany). Biotinylated horse anti-rabbit IgG secondary antibody was used (Vector BA 1000) at 1:200, and staining was revealed using a Vectastain ABC kit (Vector PK-6100) according to the manufacturer's instructions.

#### HDAC2 activity assay.

Cell lysates were prepared and subjected to HDAC immunoprecipitation as previously described (28). Immunoprecipitation was conducted with anti-HDAC2 antibody (Sigma). HDAC activity in the immunoprecipitates was assessed using a fluorometric assay kit (Biovision, Mountain View, CA). Phosphorylated levels of HDAC2 were measured using an anti-p-Ser394-HDAC2 (Abcam, Cambridge, UK).

#### Western blot.

Whole cell lysates were subjected to Western blotting as previously described (28). p-Ser473-Akt, GSK3β, phospho-GSK3β-Ser9, ERK1/2, and p-ERK1/2 antibodies were purchased from Cell Signaling Technology (Herts, UK). Anti-human β-actin was obtained from Santa Cruz Biotechnology and anti-human GAPDH antibody from Abcam.

#### GM-CSF and CXCL8 cytokine release.

Levels of GM-CSF and CXCL8 were analyzed in cell-free supernatants by sandwich ELISA (R&D Systems, Abingdon, UK) according to the manufacturer's instructions.

#### Genome-wide mRNA expression profile.

Monocytes were treated with CT99021 (1 μM), dexamethasone (10^−8^ M, 30 min), and LPS (10 ng/ml) as described above and RNA (0.5 μg) was extracted using the RNeasy Mini Kit (Qiagen, Crawley, UK). The mRNA expression profile was determined using the Agilent SurePrint G3 Human microarrays v2 following the manufacturer's instructions.

Differential gene expression was determined using the Partek Genomics Suite using a false discover rate <0.05. Differences >1.2-fold on mRNA expression were taken into consideration for our analysis. Gene sets significantly enriched in Partek were transferred to the Database for Annotation, Visualisation and Integrated Discovery (DAVID) version 6.7 (http://david.abcc.ncifcrf.gov/). Pathway analysis was performed by Kyoto Encyclopaedia of Genes and Genomes (KEGG).

#### Statistical analysis.

Data were analyzed by Friedman or Kruskal-Wallis ANOVA and the Mann-Whitney test to determine statistical significance of nonparametric data. For parametric data, ANOVA and Dunnett's posttest were used for tests between groups and the Student's *t*-test was used for tests within groups.

## RESULTS

### 

#### Increased phospho-GSK3β-Ser9 levels in COPD alveolar macrophages, monocytes, and bronchial epithelial cells.

**Fig. 1.**
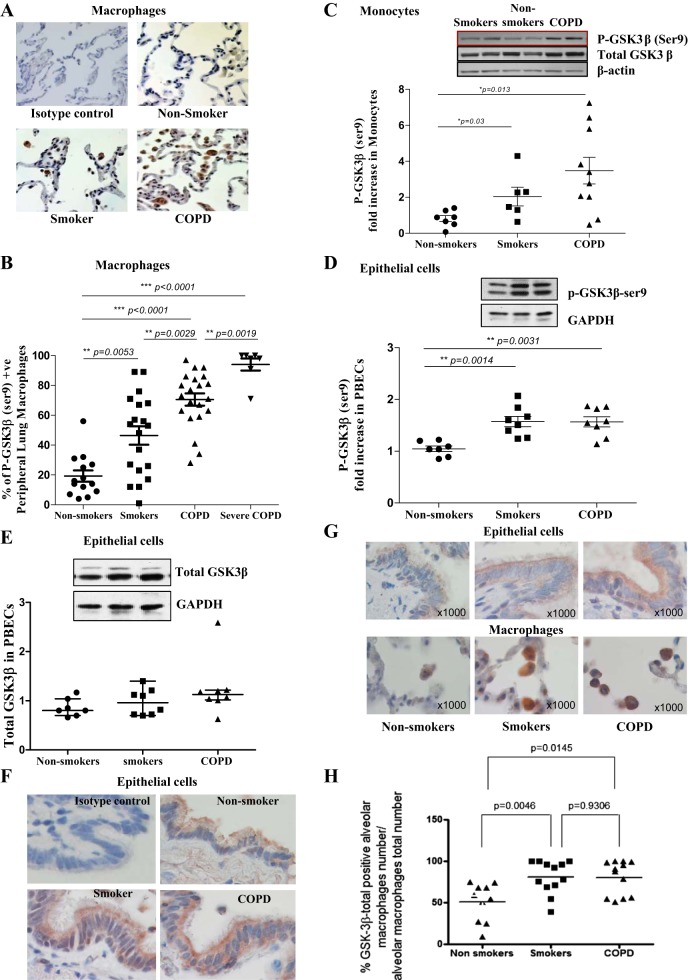
Phospho (p)-glycogen synthase 3β (GSK3β)-Ser9 levels are elevated in peripheral lung alveolar macrophages, peripheral blood monocytes, and primary bronchial epithelial cells (PBECs) from chronic obstructive pulmonary disease (COPD) patients. Representative images (*A*) and percentage of macrophages (*B*) positively stained for p-GSK3β-Ser9 in peripheral lung sections from nonsmokers (*n* = 14), smokers (*n* = 19), mild-moderate COPD (*n* = 21), and severe COPD patients (*n* = 7). Ratio of p-GSK3β/total GSK3β in primary monocytes (*n* = 6–10) with representative blots (*C*) and p-GSK3β/GAPDH (*D*) and total GSK3β/GAPDH (*E*) with representative blots in PBECs from nonsmokers, smokers, and Global Initiative for Chronic Obstructive Lung Disease (GOLD) stage IV COPD patients (*n* = 7–8). *F*: representative images of staining of p-GSK3β in large airway epithelial cells in peripheral lung sections from nonsmokers, smokers, and COPD patients are shown beneath along with an isotype-stained section as a control. *G*: total GSK3β staining in macrophages of COPD subjects and controls. *H*: percentage of GSK3β-positive macrophages. *P* values are indicated and as tested by Kruskal-Wallis ANOVA.

We first assessed the levels of phosphorylated/inactive GSK3β in peripheral lung tissue, peripheral blood monocytes and PBECs from COPD patients, nonsmokers and smokers with normal lung function. Phospho-GSK3β-Ser9 staining was higher in lung tissue macrophages of COPD patients (70.5 ± 4.1% positive) than in control smokers (46.5 ± 6.2, *P* < 0.01) and nonsmokers (19.3 ± 3.8, *P* < 0.001) and was also higher in control smokers than nonsmokers (Fig. 1, *A* and *B*). The levels of phospho-GSK3β staining were even higher in macrophages (84.9 ± 9.8% positive, *P* < 0.01) from severe COPD compared with moderate COPD patients (Fig. 1*B*). We were unable to obtain bronchoalveolar lavage (BAL) samples to confirm these results in BAL macrophages using Western blotting. The ratio of phosphorylated to total GSK3β was increased in peripheral blood monocytes from COPD patients compared with healthy subjects with no smoking history as determined by Western blot analysis (0.81 ± 0.17 vs. 3.48 ± 0.74, *P* < 0.05; Fig. 1*C*). Furthermore, phospho-GSK3β-Ser9 levels were significantly increased in PBECs from COPD stage GOLD IV patients (1.57 ± 0.10, *P* < 0.05) and control smokers (1.57 ± 0.10, *P* < 0.05) compared with nonsmoking individuals (1.05 ± 0.05; Fig. 1*D*) without significant differences in total GSK3β levels (Fig. 1*E*). Immunohistochemical staining also indicated strong phospho-GSK3β-Ser9 staining in bronchial epithelium of smokers with and without COPD, although all bronchial epithelial cells in lung tissue stained positive for phospho-GSK3β-Ser9 and we did not detect clear differences compared with nonsmoking controls using this method (Fig. 1*F*). Immunostaining for total GSK3β, detecting both phosphorylated and nonphosphorylated forms, indicated similar, albeit less strong, increases in staining in macrophages from smokers with and without COPD, without a significant difference between these two groups (Fig. 1, *G* and *H*). All bronchial epithelial cells were stained, and we did not observe differences between the groups (Fig. 1*G*). Having shown inactivation of GSK3β in different airway cells and blood monocytes in COPD patients, we examined the functional consequences of this altered activation state in monocytes/macrophages and airway epithelial cells.

#### GSK3β inhibition abrogates glucocorticoid responsiveness in primary human blood monocytes.

**Fig. 2.**
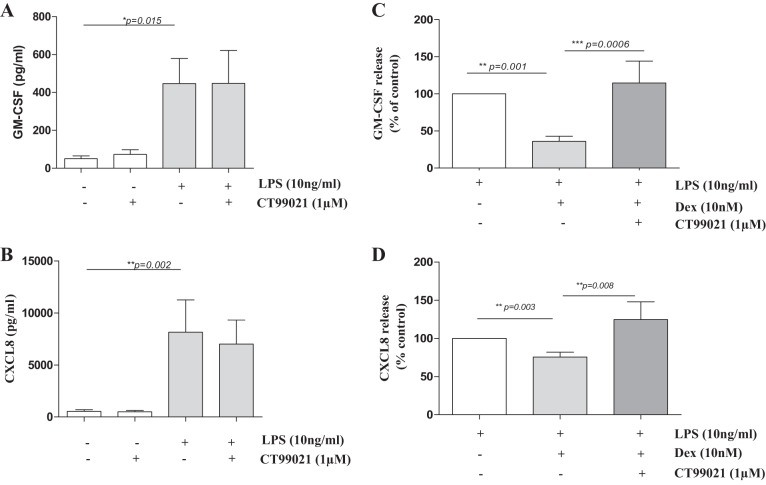
GSK3β inhibition attenuates the anti-inflammatory action of glucocorticoids in monocytes. CT99021 does not affect baseline or LPS-induced granulocyte-macrophage colony-stimulating factor (GM-CSF) or CXCL8 secretion in primary monocytes from non-COPD individuals. GM-CSF (*A*) and CXCL8 (*B*) levels were measured in supernatants of primary monocytes upon pretreatment with CT99021 for 30 min followed by 24 h of LPS. Treatment of monocytes isolated from healthy subjects with CT99021 inhibits dexamethasone (Dex)-induced suppression of LPS-stimulated (*C*) GM-CSF and (*D*) CXCL8 release (means ± SE; *n* = 6–7). *P* values are indicated.

We next investigated whether GSK3β inactivation affects the regulation of inflammatory cytokines. Treatment of monocytes from healthy subjects with the selective GSK3β inhibitor CT99021 had no effect on basal GM-CSF and CXCL8 release nor on the release upon treatment with the proinflammatory stimulus LPS (Fig. 2, *A* and *B*).

Previously, we showed that the glucocorticoid dexamethasone was less effective at repressing LPS-induced GM-CSF and CXCL8 release in blood monocytes from patients with COPD compared with age-matched smokers (27). Therefore, we also investigated the effect of GSK3β inactivation on glucocorticoid responsiveness upon LPS stimulation in monocytes. Of interest, dexamethasone (10 nM)-dependent inhibition of LPS-induced GM-CSF (63.9 ± 6.7%, *P* < 0.05) and CXCL8 (24.42 ± 6.5%, *P* < 0.05) release was completely abrogated by CT99021 (Fig. 2, *C* and *D*).

#### Oxidative stress induces PI3K/Akt-dependent inhibition of GSK3β activity in monocytes.

**Fig. 3.**
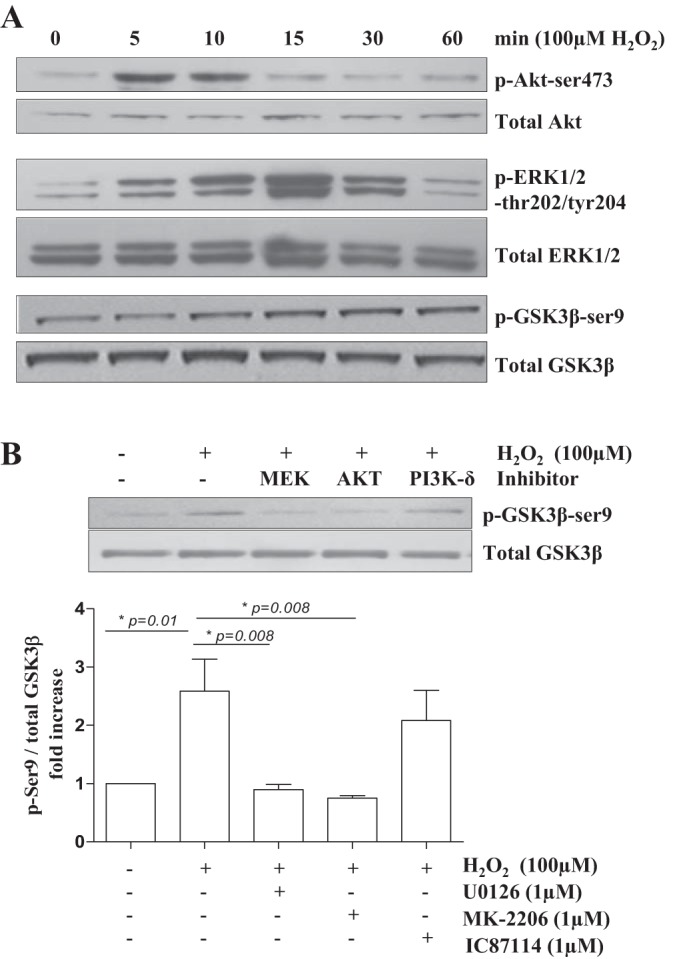
Oxidative stress induced inactivation of GSK3β is mediated via phosphatidylinositol 3-kinase (PI3K)/Akt in primary monocytes. *A*: primary monocytes from non—–COPD individuals were exposed to H_2_O_2,_ which time-dependent increase in PI3K, ERK1/2, and GSK3β phosphorylation as detected by Western blotting. Representative blots of 4 independent experiments are shown. *B*: GSK3β phosphorylation is PI3K/Akt and ERK1/2-dependent in monocytes as indicated by pretreatment of the cells with the MEK/ERK-1/2 inhibitor U0126, the Akt inhibitor MK-2206, and the PI3Kδ inhibitor IC87114. Densitometry was performed and p-GSK3β-Ser9 levels are expressed as ratio of total GSK3β (means ± SE; *n* = 5).

Because of our hypothesis that oxidative stress induces glucocorticoid responsiveness, we next examined how GSK3β activity is modulated in response to exogenous reactive oxygen species (ROS) in primary monocytes, as both H_2_O_2_ and CSE exposure reduce glucocorticoid sensitivity in monocytes (7, 30, 31). H_2_O_2_ increased phospho-GSK3β-Ser9 levels in a time-dependent manner (Fig. 3*A*). Exposure to H_2_O_2_ also activated PI3K as measured by increased levels of phospho-Akt-Ser473, at earlier time points than GSK3β-Ser9 phosphorylation (Fig. 3*A*). Inhibition of Akt (MK-2206) reversed the oxidant-induced inactivation of GSK3β (Fig. 3*B*). Similarly, GSK3β phosphorylation was reduced by the MEK/ERK-1/2 inhibitor U0126, indicating involvement of the ERK1/2 pathway in the oxidant-induced effect on GSK3β in primary monocytes (Fig. 3, *A* and *B*). We previously showed that PI3Kδ is responsible for the oxidant-induced activation of Akt in monocytes (27). However, selective inhibition of PI3Kδ with IC87114 did not affect oxidant induction of phospho-GSK3β-Ser9 in monocytes (Fig. 3*B*), indicating involvement of other PI3K isoforms or signaling mediators.

#### GSK3β protein knockdown and overexpression of inactive GSK3β reduce glucocorticoid function in monocytes.

**Fig. 4.**
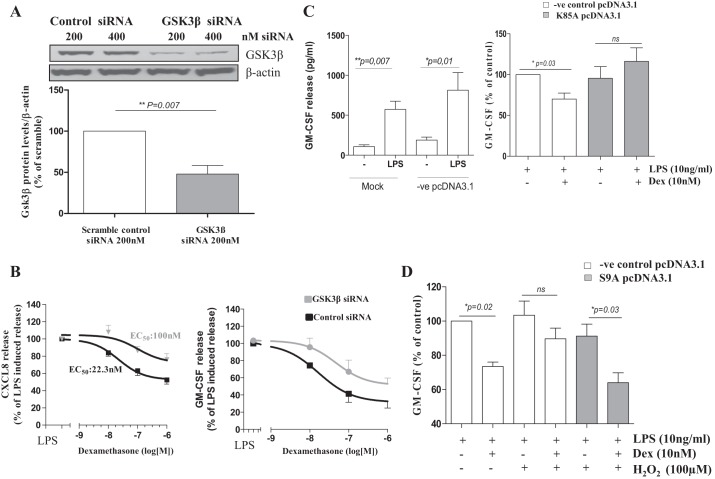
GSK3β modulates dexamethasone function in MonoMac6 cells. *A*: GSK3β levels are reduced by GSK3β siRNA after 24 h of transfection. Densitometry was performed and GSK3β levels are expressed as ratio of β-actin as loading control. *B*: GSK3β siRNA knockdown (24 h) inhibits the concentration-dependent suppression of LPS-induced CXCL8 (*left*) and GM-CSF (*right*) release by dexamethasone (means ± SE; *n* = 6). *C*: LPS-induced GM-CSF release in mock transfected monocytes and cells transfected with the positive control pcDNA3.1 (pg/ml; means ± SE; *n* = 4), and effect of overexpression of the inactive mutant GSK3βK85A on dexamethasone suppression of LPS-induced GM-CSF release (%; means ± SE; *n* = 4). *D*: overexpression of the constitutively active GSK3βS9A mutant restores H_2_O_2_-induced dexamethasone unresponsiveness of GM-CSF release (means ± SE; *n* = 4).

To gain further mechanistic insight in the role of GSK3β in oxidant-induced glucocorticoid unresponsiveness in monocytes, we used siRNA to knockdown total GSK3β levels and analyze the anti-inflammatory actions of dexamethasone. Transfection of MonoMac6 cells with GSK3β on-target siRNA significantly reduced GSK3β total protein levels compared with scrambled control siRNA (Fig. 4*A*). In line with the CT99021 effect, knockdown of GSK3β significantly inhibited the ability of dexamethasone to suppress CXCL8 expression, and the dexamethasone EC_50_ was increased from 22 to 100 nM. In addition, the inhibitory effect of dexamethasone on LPS-stimulated CXCL8 expression was decreased from 52.5 ± 4.8 to 75.8 ± 7.1% (Fig. 4*B*, *left*). Similar effects were seen with GSK3β knockdown on the dexamethasone suppression of LPS-induced GM-CSF secretion (Fig. 4*B*, *right*).

To validate our findings, we overexpressed the K85A kinase dead GSK3β mutant and analyzed dexamethasone function. MonoMac6 and primary cells produce similar levels of inflammatory mediators following stimulation with LPS (Fig. 4*C*, *left*). Dexamethasone significantly reduced LPS-induced GM-CSF release by 33.1 ± 2.8% in MonoMac6 cells transfected with the control pcDNA3.1. Dexamethasone had no significant inhibitory effect on LPS-induced GM-CSF release when the K85A GSK3β mutant was overexpressed (91.3 ± 12.2% vs. 111.4 ± 15.5%, Fig. 4*C*).

To confirm that GSK3β mediates oxidant-induced glucocorticoid insensitivity in monocytes, we overexpressed a constitutively active S9A GSK3β mutant in MonoMac6 cells and analyzed dexamethasone function during H_2_O_2_ exposure. In line with previous studies (27), the anti-inflammatory effect of dexamethasone was significantly attenuated by H_2_O_2_ pretreatment (Fig. 4*D*). In the presence of the active S9A mutant, H_2_O_2_-induced dexamethasone insensitivity was suppressed, leading to a significant (27.2 ± 1.3%) inhibition of LPS-stimulated GM-CSF release, similar to that observed in control cells (26.7 ± 2.5% inhibition; Fig. 4*D*).

#### GSK3β-regulated glucocorticoid responsiveness is HDAC2-dependent in human monocytes.

**Fig. 5.**
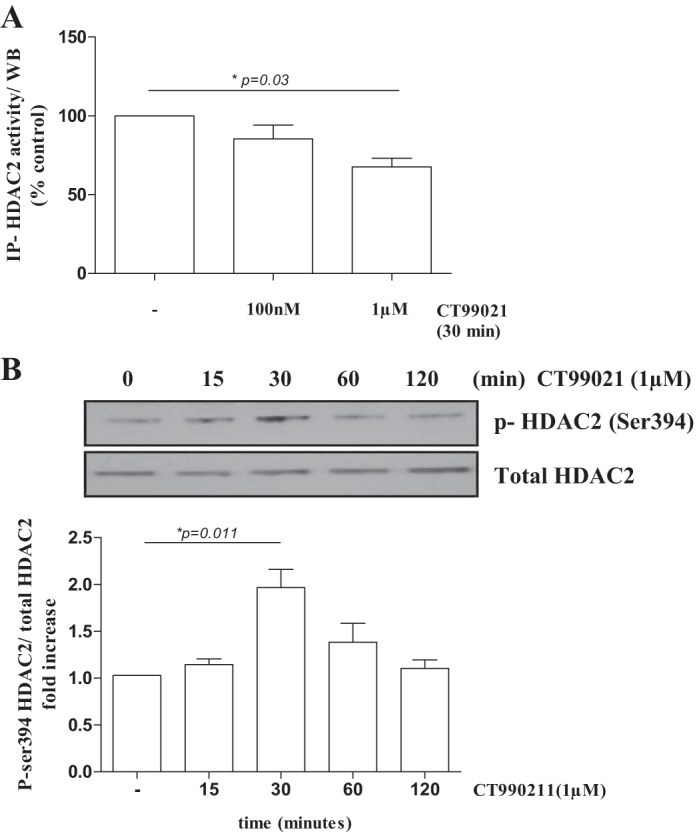
GSK3β-regulated glucocorticoid function is histone deacetylase 2 (HDAC2)-dependent in monocytes. *A*: treatment of primary monocytes with CT99021 inhibits the enzymatic activity of HDAC2 (means ± SE; *n* = 4). *B*: CT99021 treatment induces p-HDAC2-Ser394 in primary monocytes. Densitometry was performed and p-HDAC2 levels are expressed as ratio of total HDAC (means ± SE; *n* = 4). IP, immunoprecipitation; WB, Western blot.

Since HDAC2 has been implicated in oxidative stress-induced glucocorticoid unresponsiveness (4), we investigated whether ROS-induced inactivation of GSK3β may lead to modulation of HDAC2 activity in primary monocytes. Inhibition of GSK3β activity by treatment with CT99021 reduced the activity of immunoprecipitated HDAC2 (Fig. 5*A*). This reduction in HDAC2 activity correlated with increased phosphorylation of serine 394 (Fig. 5*B*), while HDAC2 mRNA and protein levels were not affected by GSK3β inactivation (data not shown).

#### Effect of GSK3β inhibition on dexamethasone-regulated inflammatory gene expression in human monocytes.

We also investigated the effect of CT99021 on dexamethasone regulation of LPS-induced gene expression using gene arrays. Partek analysis identified 17 genes that were differentially expressed (false discovery rate <0.05) upon CT99021 exposure. CT99021 specifically affected genes involved in the Wnt/β-catenin signaling pathway (*P* = 0.05) in LPS-stimulated MonoMac6 cells (data not shown), thereby confirming the specificity of CT99021 action. We next investigated the effect of CT99021 on LPS/dexamethasone-treated monocytes. On-hundred and sixty-four known genes were differentially expressed upon GSK3β inhibition in the presence of LPS/dexamethasone (*P* < 0.05). KEGG analysis showed key pathways that these genes regulate (Table 5). Thirteen out of the 164 genes encode for inflammatory chemokines (such as CXCL6, CXCL3, CXCL2, and CXCL1), cytokines (such as GM-CSF and G-CSF) and cytokine/chemokine receptors involved in the cytokine-cytokine receptor interaction pathway (*P* = 2.4 × 10^−5^). Ten more genes encode for proteins involved in chemokine signaling pathways (*P* = 2 × 10^−4^), and seven genes encode for proteins that regulate the neuro-active receptor-ligand interaction (*P* = 6.5 × 10^−2^).

**Table 5.** Pathways affected by differential gene expression in response to CT99021 treatment in MonoMac6 cells

Category	Term	Genes (out of 164)	%Total Number Benes	*P* Value
KEGG_PATHWAY	Cytokine-cytokine receptor interaction	13	7.9%	2.4E-5
KEGG_PATHWAY	Chemokine signaling pathway	10	6.1%	2.0E-4
KEGG_PATHWAY	Neuroactive ligand-receptor interaction	7	4.2%	6.5E-2

Kyoto Encyclopaedia of Genes and Genomes (KEGG) analysis showed 164 known genes that were differentially expressed due to GSK3β inhibition in the presence of LPS/dexamethasone. Thirteen out of the 164 genes encode for inflammatory chemokines. Ten genes encode for proteins involved in chemokine signaling pathways and 7 genes encode for proteins that regulate the neuroactive receptor-ligand interaction.

#### GSK3β inhibition abrogates glucocorticoid responsiveness in primary 16HBE cells.

**Fig. 6.**
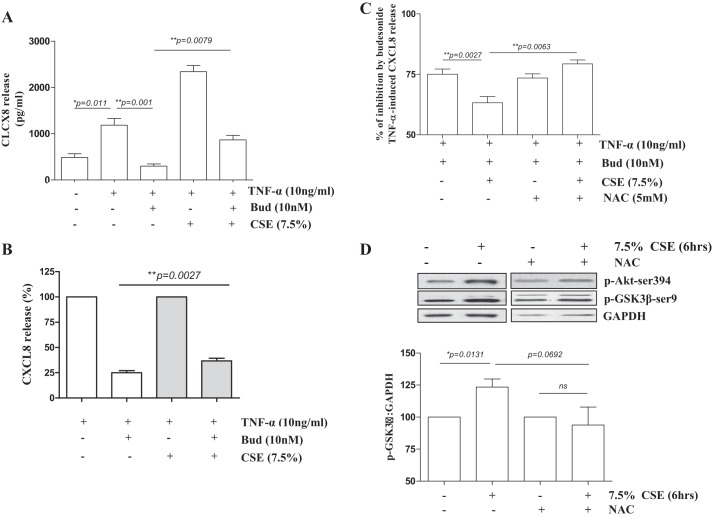
GSK3β inhibition attenuates the anti-inflammatory action of glucocorticoids in primary bronchial epithelial cells (PBECs). CT99021 does not affect GM-CSF or CXCL8 secretion in PBECs. GM-CSF (*A*) and CXCL8 (*B*) levels were measured in cell-free supernatants of PBECs from non-COPD individuals upon pretreatment with CT99021 for 30 min followed by 24 h TNF-α stimulation of PBECs (means ± SE; *n* = 4–6). Pretreatment with CT99021 (30 min) reverses budesonide (Bud)-induced suppression of TNF-α-stimulated GM-CSF (*C*) and CXCL8 (*D*) release in PBECs from non-COPD individuals (means ± SE; *n* = 8).

We previously reported that TNF-α-induced GM-CSF production in PBECs from GOLD stage II COPD patients was less responsive to the clinically used inhaled glucocorticoid budesonide compared with nonsmoking controls, with an intermediate effect of budesonide in PBECs from control smokers (16). In a similar manner to monocytes, CT99021 had no effect on GM-CSF and CXCL8 release in PBECs from non-COPD individuals at baseline or upon stimulation with TNF-α, a relevant mediator of inflammation in COPD (Fig. 6, *A* and *B*). In further line with our findings in monocytes, pretreatment of PBECs from non-COPD individuals with CT99021 resulted in complete abrogation of the anti-inflammatory effect of budesonide on TNF-α-stimulated GM-CSF and CXCL8 release (Fig. 6, *C* and *D*).

#### Oxidative stress leads to reduced glucocorticoid responsiveness, activation of the PI3K/Akt pathway, and inhibition of GSK3β activity in bronchial epithelial cells.

**Fig. 7.**
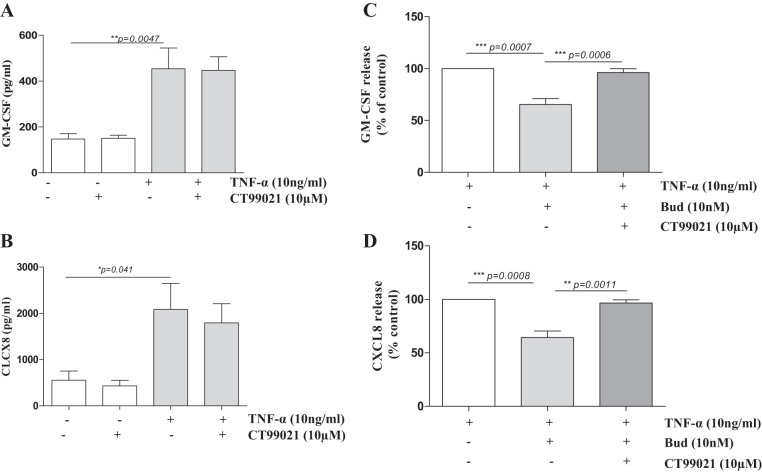
Cigarette smoke extract (CSE)-induced oxidative stress induces budesonide unresponsiveness in TNF-α-stimulated 16HBE cells. *A*–*C*: pretreatment with CSE reduces budesonide-induced suppression of TNF-α-stimulated CXCL8 release and pretreatment with *N*-acetyl-cysteine (NAC; 30 min) restores CSE-induced budesonide unresponsiveness (means ± SE; *n* = 5). Absolute values (*A*), values related to the TNF-α-induced control (*B*) and percent inhibition by budesonide (*C*) are shown (*D*) CSE induces PI3K and GSK3β phosphorylation cells, as indicated by Western blotting. Representatives of 3 independent experiments are shown. The CSE-induced increase in GSK3β phosphorylation is abrogated by NAC pretreatment. Densitometry was performed and p-GSK3β-Ser9 levels are expressed as ratio of GAPDH as loading control (means ± SE; *n* = 6).

Because of the limited cell numbers of primary cultures, further mechanistic studies were performed in the human bronchial cell line 16HBE. Bronchial epithelial cells are in direct contact with inhaled cigarette smoke, which known to induce oxidative stress in these cells (4, 33, 41). Therefore, we studied the effect of CSE, which is known to exert similar effects in 16HBE cells and PBECs at least in part through oxidative stress mechanisms (18). Exposure of 16HBE cells to CSE (7.5%) for 6 h led to a significant upregulation of HO-1 mRNA, a marker of oxidative stress, which was blocked by the oxidant scavenger NAC, confirming that CSE exposure induces oxidative stress in epithelial cells (data not shown). In addition, CSE exposure reduced the ability of budesonide to suppress TNF-α-induced CXCL8 release (Fig. 7, *A* and *B*), which was reversed by NAC, confirming the involvement of oxidative stress (Fig. 7*C*). Similar to the effects of oxidative stress in monocytes, CSE (7.5%) induced activation of PI3K/Akt signaling and inactivation of GSK3β in 16HBE cells, which was no longer present upon treatment with NAC, although the difference between CSE and CSE + NAC did not reach significance (*P* = 0.0692). Together, our data show that CSE-derived ROS result in GSK3β inactivation in bronchial epithelial cells and that GSK3β inhibition reduces glucocorticoid sensitivity of proinflammatory responses in both human monocytes and bronchial epithelial cells.

Together, our data indicate that GSK3β inhibition reduces glucocorticoid sensitivity of proinflammatory responses in both human monocytes and bronchial epithelial cells without modulating inflammatory mediator expression per se. The mechanism for the effect differs between cell types.

## DISCUSSION

The increased oxidant burden derived from cigarette smoking in the lungs of COPD patients has been associated with reduced glucocorticoid responsiveness. The molecular mechanisms of oxidative stress-induced glucocorticoid unresponsiveness have remained unknown to date. Our data show, for the first time, that levels of inactive phosphorylated GSK3β are higher in lung macrophages, peripheral blood monocytes, and bronchial epithelial cells from COPD patients compared with control subjects. In both monocytes and bronchial epithelial cells, pharmacological inactivation of GSK3β resulted in reduced responsiveness of inflammatory mediators to glucocorticoids. We observed a difference in glucocorticoid insensitivity between nonsmoking controls and COPD patients but not between current smokers and COPD patients or current smokers and healthy controls. This indicates that smokers have an intermediate state of sensitivity and that individuals with COPD are more susceptible to develop steroid insensitivity upon smoking. Furthermore, we previously observed more pronounced glucocorticoid unresponsiveness in epithelial cells from severe compared with moderate COPD patients (16). Because severe COPD patients are dependent on the use of inhaled or oral glucocorticoids, it is of importance to elucidate the mechanisms of glucocorticoid unresponsiveness in COPD to improve the treatment of patients with severe symptoms.

Since we observed that phospho-GSK3 levels were still increased in PBECs from severe, ex-smoking COPD patients, even after 2–3 wk of culture, we anticipate that there may be persistent alterations in the regulators of GSK3 phosphorylation, resulting from rewiring of the intracellular inflammatory pathways rather than an effect of recent exposure to the local inflammatory milieu in the COPD lung. The antibody used to detect GSK3β also detects GSK3α; thus although we cannot discount a role of GSK3α here, they have identical functions. With respect to therapeutic intervention, it will be important to further elucidate the downstream mechanisms involved in the reduced glucocorticoid responsiveness upon GSK3β inactivation. GSK3β is involved in numerous intracellular pathways, and preventing its inactivation, e.g., by pharmacological inhibition of the PI3K/Akt or MAPK pathways, may lead to serious side effects.

In line with our results, the activation of GSK3β has been previously implicated in glucocorticoid-induced apoptosis in lymphoma cells, its inactivation resulting in glucocorticoid resistance, although effects on inflammatory responses were not studied (35). In monocytes, GSK3β inactivation reduced glucocorticoid suppression of proinflammatory responses by inhibition of the enzymatic activity of HDAC2. This reduction in activity was not associated with a change in expression but an increase in HDAC2 phosphorylation at serine 394. Casein kinase 2 (CK2) phosphorylates HDAC2 at serine 394 and is a direct target of GSK3β, negatively regulating its function (38). Therefore, inactivation of GSK3β in monocyte-macrophages may increase CK2-induced phosphorylation of HDAC2. This may be involved in the observed glucocorticoid unresponsiveness towards NF-κB-induced proinflammatory cytokine production upon GSK3β inactivation in monocytes/macrophages. Although the functions of individual HDAC2 phosphorylation sites and the responsible kinases are unclear, the activity of this important GRα corepressor may deprive GRα of a key mechanism by which to control inflammatory gene expression.

In line with our findings in monocytes, our data indicate that glucocorticoid unresponsiveness is induced upon GSK3β inactivation in airway epithelial cells and that cigarette smoke-induced oxidative stress may be responsible for this effect. Our combined data from monocytes and epithelial cells suggest that GSK3β may be an important common redox sensing effector molecule for a number of signaling pathways including MEK/ERK-1/2 and PI3K/Akt, regulating NF-κB activation, and the subsequent inflammatory mediator release and inflammatory cell recruitment (12, 29). These redox sensitive p38 MAPK, ERK-1/2, and PI3K/Akt pathways can all induce GSK3β phosphorylation (8, 15) and have been implicated in glucocorticoid insensitivity in COPD (5). This further corroborates the role of GSK3β in oxidative stress-induced glucocorticoid unresponsiveness.

The monocyte microarray data show that the anti-inflammatory effects of dexamethasone are prevented in the presence of CT99021. Not surprisingly, therefore, the effect of GSK3β inhibition on enhancing the expression of LPS-induced inflammatory genes was more marked in the presence of dexamethasone. These genes, mostly chemokines and cytokines, regulate signaling pathways that induce neutrophil, lymphocyte, and macrophage activation indicating that GSK3β activity is important for regulating multiple inflammatory pathways modulated by glucocorticoids. This supports our hypothesis that aberrant GSK3β activity is involved in driving chronic inflammation through enhancing glucocorticoid insensitivity in COPD.

GSK3β inhibition also resulted in significant hits in the neuropeptide/neurotransmitter receptor interacting pathways, including those for the 5-hydroxytryptamine (5-HT) receptors 2b and 6 (HTR2b and HTR6), the protease-activated receptor (PAR) family, galanin receptor 2 (Galr2), the glutamate receptor delta 2 (Grid2), and GABA B receptors 1 and 2. The neurotransmitter serotonin (5-HT) is also released from the neuroendocrine cells of the human lung, increasingly recognized for their immunomodulatory effects outside the central nervous system and their contribution to the pathogenesis of autoimmune and chronic inflammatory diseases (40). The serum concentration of the tryptophan, the amino acid precursor of the 5-HT, is increased in patients with COPD (42), whereas plasma 5-HT levels are elevated in smokers with normal lung function but reduced in COPD patients (39). In addition, cigarette smoke affects airway hyperresponsiveness through 5-HT in precision-cut lung slices (11). Finally, recent evidence indicates that 5-HT suppresses efferocytosis in human alveolar macrophages, although this appears to be independent of HTR2B (40). COPD patients have higher maximum thrombin levels, rates of thrombin generation, and total thrombin formation although this was not linked to severity or inflammatory mediator expression (43). PAR-1 is activated by the thrombin, and it is overexpressed in the alveolar macrophages from smokers with normal lung function (34). PAR-4 is activated by thrombin and trypsin. PAR-4 methylation and altered expression may be important in the enhanced risks associated with cigarette smoking that continue even after cessation (45). GABA is produced by the bronchial epithelium and contributes to the relaxation of airway smooth muscle tone (14). GABA_A_ receptors are known to be expressed on bronchial epithelial cells, mediating mucus production in response to nicotine (13), while the loss of GABA_B_ receptors modifies the biochemical and behavioral responses to nicotine withdrawal (44a). However, the function of GABA_B_ receptors in the human airways and in COPD patients is unknown. It is increasingly evident that there are neural-like transmitter interactions in human bronchial epithelial cells, which may link central nervous system-active drugs to the increased inflammation and mucin production seen in COPD.

Taken together, our study shows that reduced GSK3β activity in COPD monocytes-macrophages and airway epithelial cells may contribute to cigarette smoke-induced glucocorticoid insensitivity in the airways of COPD patients. The key nodal function of GSK3β in integrating various ROS-induced upstream and downstream signaling pathways in different cell types suggests that reversing this inactivation may constitute a novel therapeutic strategy to improve glucocorticoid function and thereby suppress airway inflammation in COPD.

## GRANTS

This research was partially performed within the framework of the Top Institute Pharma Project T1-201 “COPD, Transition of Systemic Inflammation into Multi-Organ Pathology,” with partners University Medical Center Groningen, University Medical Center Utrecht, University Medical Center Maastricht, Nycomed, GlaxoSmithKline, Danone, AstraZeneca, and Foundation TI Pharma. This project was supported by the National Institute for Health Research Respiratory Biomedical Research Unit at the Royal Brompton and Harefield National Health Service Foundation and Imperial College London. A. Ngkelo is the recipient of a Biotechnology and Biological Sciences Research Council CASE award with Pfizer. I. M. Adcock and P. J. Barnes are supported by a Wellcome Trust programme grant and through COPD-MAP. G. Caramori is supported by unrestricted educational grants from AstraZeneca Italy, Boehringer Ingelheim Italy, GSK Italy, and Menarini.

## DISCLOSURES

No conflicts of interest, financial or otherwise are declared by the author(s).

## AUTHOR CONTRIBUTIONS

Author contributions: A.N., R.F.H., G.C., F.M., F.A., G.S., K.F.C., A.P., N.H.t.H., P.J.B., A.J.M.v.O., I.M.A., P.A.K., and I.H.H. conception and design of research; A.N., R.F.H., A.L.D., J.A.M., S.M.B., H.G.d.B., M.R.J., C.R., E.T., M.C., P.C., F.M., F.A., G.S., I.K., M.A.L., M.v.d.B., and I.H.H. performed experiments; A.N., R.F.H., A.L.D., J.A.M., S.M.B., H.G.d.B., M.R.J., E.T., G.C., M.C., P.C., l.k., K.F.C., A.P., M.A.L., W.T., I.M.A., P.A.K., and I.H.H. analyzed data; A.N., R.F.H., A.L.D., J.A.M., S.M.B., H.G.d.B., M.R.J., E.T., G.C., M.C., F.M., F.A., G.S., I.K., K.F.C., A.P., M.A.L., N.H.t.H., M.v.d.B., W.T., P.J.B., A.J.M.v.O., I.M.A., P.A.K., and I.H.H. interpreted results of experiments; A.N., R.F.H., S.M.B., H.G.d.B., C.R., P.C., I.M.A., and I.H.H. prepared figures; A.N., R.F.H., A.J.M.v.O., I.M.A., P.A.K., and I.H.H. drafted manuscript; A.N., R.F.H., I.K., K.F.C., A.P., N.H.t.H., P.J.B., A.J.M.v.O., I.M.A., P.A.K., and I.H.H. edited and revised manuscript; A.N., R.F.H., A.L.D., J.A.M., S.M.B., H.G.d.B., M.R.J., C.R., E.T., G.C., P.C., F.M., F.A., G.S., I.K., K.F.C., A.P., M.A.L., N.H.t.H., M.v.d.B., W.T., P.J.B., A.J.M.v.O., I.M.A., P.A.K., and I.H.H. approved final version of manuscript.
